# PIRSF Family Classification System for Protein Functional and Evolutionary Analysis

**Published:** 2007-02-10

**Authors:** Anastasia N. Nikolskaya, Cecilia N. Arighi, Hongzhan Huang, Winona C. Barker, Cathy H. Wu

**Affiliations:** Protein Information Resource, Department of Biochemistry and Molecular & Cellular Biology

**Keywords:** Domain architecture, Functional convergence, Functional divergence, Genome context, Protein family classification, Taxonomic distribution

## Abstract

The PIRSF protein classification system (http://pir.georgetown.edu/pirsf/) reflects evolutionary relationships of full-length proteins and domains. The primary PIRSF classification unit is the homeomorphic family, whose members are both homologous (evolved from a common ancestor) and homeomorphic (sharing full-length sequence similarity and a common domain architecture). PIRSF families are curated systematically based on literature review and integrative sequence and functional analysis, including sequence and structure similarity, domain architecture, functional association, genome context, and phyletic pattern. The results of classification and expert annotation are summarized in PIRSF family reports with graphical viewers for taxonomic distribution, domain architecture, family hierarchy, and multiple alignment and phylogenetic tree. The PIRSF system provides a comprehensive resource for bioinformatics analysis and comparative studies of protein function and evolution. Domain or fold-based searches allow identification of evolutionarily related protein families sharing domains or structural folds. Functional convergence and functional divergence are revealed by the relationships between protein classification and curated family functions. The taxonomic distribution allows the identification of lineage-specific or broadly conserved protein families and can reveal horizontal gene transfer. Here we demonstrate, with illustrative examples, how to use the web-based PIRSF system as a tool for functional and evolutionary studies of protein families.

## Introduction

High-throughput genome projects have resulted in a rapid accumulation of predicted protein sequences. To fully realize the value of the data, scientists need to understand how these proteins function in making up a living cell. With experimentally verified information on protein function lagging far behind, computational methods are needed for reliable and large-scale functional annotation of proteins. A general approach for functional characterization of unknown proteins is to infer protein functions based on sequence similarity to annotated proteins in sequence databases. While this is a powerful approach that has led to many scientific discoveries, numerous genome annotation errors have been detected (Devos and Valencia 2001), many of which have been propagated throughout other molecular databases.

Classification of proteins is widely accepted to provide valuable clues to structure, function and evolution. Protein family classification has several advantages as a basic approach for large-scale annotation: (i) it improves the annotation of proteins that are difficult to characterize based on pair-wise alignments; (ii) it assists database maintenance by promoting family-based propagation of annotation and making annotation errors apparent; (iii) it provides an effective means to retrieve relevant biological information from vast amounts of data; and (iv) it reflects the underlying gene families, the analysis of which is essential for comparative genomics and phylogenetics.

To facilitate accurate, consistent and rich functional annotation of proteins, the Protein Information Resource (PIR, http://pir.georgetown.edu/) employs a classification-driven annotation method supported by a bioinformatics framework that provides data integration and associative analysis. This paper describes the PIRSF family classification and functional annotation approaches and illustrates how manually curated protein families can be used to support protein functional and evolutionary studies via the PIRSF web site at http://pir.georgetown.edu/pirsf/.

## PIRSF Family Classification and Annotation

### PIRSF classification concept

Derived originally from the protein superfamily concept articulated by Margaret Dayhoff (1976), the PIRSF family classification system applies a network structure for protein classification from superfamily to subfamily levels (Wu et al 2004a). The primary PIRSF classification unit is the *homeomorphic family* whose members are *homologous* (sharing common ancestry) and *homeomorphic* (sharing full-length sequence similarity with common domain architecture). Common domain architecture is indicated by the same type, number, and order of core domains, although variation may exist for repeating domains and/or auxiliary domains. Basing classification on full-length proteins allows annotation of biological functions, biochemical activities, and sequence features that are family specific, while the domain architecture of a protein provides insight into general functional and structural properties, as well as into complex evolutionary mechanisms.

Each protein can be assigned to only one homeomorphic family, which may have zero or more parent *superfamilies* and zero or more child *subfamilies.* The parent superfamilies connect related families and orphan proteins based on one or more common domains, which may or may not extend over the entire lengths of the proteins. The child subfamilies are homeomorphic groups that may represent functional specialization. The flexible number of parent-child levels from superfamily to subfamily reflects natural clusters of proteins with varying degrees of sequence conservation. While a protein will belong to one and only one homeomorphic family, multi-domain proteins may belong to multiple superfamilies (hence, the network structure). A domain superfamily, which consists of all proteins that contain a particular domain, is usually represented by the corresponding Pfam domain (Bateman et al 2004) for convenience.

### PIRSF classification and curation workflow

**Figure 1:**
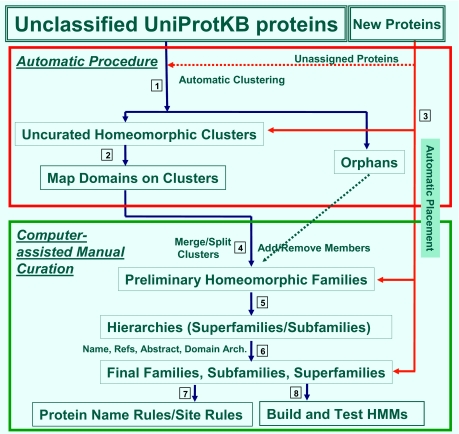
PIRSF protein family classification and curation workflow

The workflow for PIRSF family classification and curation is depicted in Figure 1. Homologous protein families are defined systematically in an iterative mode that couples manual analysis with computer-assisted clustering and information retrieval. The procedure that progresses from unclassified proteins to non-curated clusters (steps 1–3) to fully curated PIRSFs (steps 4–8) is summarized below:

1. *Computational generation of homeomorphic clusters* based on full-length sequence similarity using both pair-wise and cluster-based parameters.
2. *Computational preprocessing and domain mapping of preliminary clusters* by retrieving relevant information for all member proteins, including related sequences, sequence features (domains, motifs, sites) and selected annotation from the PIR data warehouse.
3. *Automatic placement of new* proteins into existing families based on BLAST and HMM results with stringent threshold values to avoid false positives. Assignments not made automatically can be added in Step 4.
4. *Computer-assisted expert analysis to define homeomorphic families* based on sequence similarity, domain architecture, and taxonomic distribution. Family membership is defined, delineating full members and associate members, and selecting representative members and seed members.
5. *Hierarchies (parent superfamilies and/or child subfamilies)* are created when necessary. The number of hierarchical levels varies, depending on the diversity of the protein group, evolutionary age of the subgroups and the level of functional specialization and diversity. Subfamilies are created when necessary to account for functional divergence and to provide accurate protein annotation.
6. *Expert annotation* includes extensive review of relevant publications in order to assign accurate and up-to-date names and functions to the family and its members. In the absence of experimental data, functional predictions inferred from sequence and/or structural similarity, genome context, and other evidence are made whenever possible. Name, bibliography and an optional abstract are assigned to the PIRSFs.
7. To ensure accurate and appropriate transfer of the annotations from the curated PIRSF family onto its individual member proteins, name rules and optional site rules are created.

Seed members are used for the automatic generation (with optional expert review and analysis) of family-specific hidden Markov models (HMMs), multiple sequence alignment, and neighbor-joining phylogenetic tree.

The PIRSF system consists of two data sets: noncurated clusters and curated families. Currently, about a third of UniProtKB sequences are classified into over 35,000 clusters, including single-member clusters. The non-curated clusters are computationally defined using both pairwise-based parameters and cluster-based parameters. Systematic family curation is being conducted in a two-tier process to improve the quality of automated classification, with over 4,500 preliminarily curated and 3,900 fully curated families currently available. The preliminary curation provides membership and domain architecture characteristic of the family, while the full curation provides additional annotation, including family name, parent-child relationships, family description, and bibliography. Literature-based curation ensures that users are provided with high quality, accurate and up-to-date experimental data.

### Integrative functional annotation of PIRSF families

Systematic PIRSF family curation integrates various types of information about the protein family and its members, including sequence and structure similarity, domain architecture, function, genome context, and phyletic pattern, depending upon the special properties of the protein families.

#### Sequence similarity

It is widely known that protein function can remain conserved in related proteins across major taxonomic groups and/or when sequences have diverged so that sequence similarity is very low. This allows similarity-based predictions of functions for uncharacterized proteins and protein families, ranging from fairly obvious to those requiring elaborate sequence and structure comparisons using additional tools such as PSI-BLAST, profile searches, manual construction of sequence alignments and examination of conserved residues and motifs, structure-structure alignments, and other methods.

#### Domain architecture

Protein families that contain the same domains need to be considered together in order to delineate a consistent classification and to facilitate similarity-based predictions. This approach supports better understanding of higher-order relationships among PIRSF homeomorphic families and the divergence of families/superfamilies with a given domain. Furthermore, the evolutionary mobility of certain domains (resulting in domain accretion in multi-domain proteins), the rapid sequence divergence associated with reallocation of functions, and the emergence of distinct functions among relatively close members of a protein family come into focus (Aravind et al 2002). Protein domain organization is particularly informative for analyzing multi domain protein families.

#### Function and genomic context

Surveying proteins and protein families participating in the same biochemical pathway, protein complex, or other functional subsystem facilitates annotation of the relevant proteins, especially when combined with the genomic co-localization information. In its simplest and most widely used form, context analysis means “operon structure” where, in prokaryotes, genes encoding enzymes involved in the same metabolic pathway often cluster together in the genome. Analysis of other types of context information, including protein fusion events, occurrence profiles or signatures, and shared regulatory sites can allow inference of functional coupling for proteins participating in related cellular processes. We have adopted the SEED platform developed by the FIG group (http://www.figresearch.com/) for genome context visualization to facilitate functional subsystem analysis (Osterman and Overbeek 2003).

#### Phyletic pattern

With the underlying taxonomic information, one can derive phyletic (phylogenetic) patterns of PIRSFs, indicating the presence or absence of corresponding proteins in completely sequenced genomes, to identify PIRSFs that occur only in given lineages or share common phylogenetic patterns. The latter sets of PIRSFs may indicate participation in the same functional system, especially if the associated pattern is unusual. Combining phylogenetic pattern and biochemical pathway information for protein families allows identification of cases where known functions have yet to be aligned with particular proteins. It may also allow for identifying alternative pathways for the same end product in different taxonomic groups (Wu et al 2004b).

#### Integrative annotation of unknown proteins

The collective use of multiple approaches often leads to functional prediction for families of uncharacterized proteins. The following example shows the annotation of several “conserved hypothetical” protein groups as the subunits of the [NiFe]-hydrogenase-3-type complex Eha, based on co-expression data, sequence conservation, genome context and phyletic profile.

The energy-converting hydrogenase A (*eha*) operon encodes a putative multisubunit membrane-bound [NiFe]-hydrogenase Eha in *Methanobacterium thermoautotrophicum* (Tersteegen and Hedderich 1999). Experimental data on transcription suggests that the *eha* operon encodes at least 20 proteins (Tersteegen and Hedderich 1999), including four broadly conserved [NiFe]-hydrogenase subunits: large subunit (PIRSF000230, subfamily PIRSF500033), small subunit (PIRSF002913, subfamily PIRSF500034), membrane subunit J (PIRSF000215, subfamily PIRSF500037), and an integral membrane protein (PIRSF036536) that shares sequence similarity to the N-terminal half of the [NiFe]-hydrogenase large membrane subunit (Table 1). These four proteins show high sequence similarity to subunits of the Ech hydrogenase from *Methanosarcina barkeri*, hydrogenases 3 and 4 (Hyc and Hyf) from *Escherichia coli*, and CO-induced hydrogenase (Coo) from *Rhodospirillum rubrum*, all of which form a distinct group of multisub-unit membrane-bound [NiFe]-hydrogenases and together are called hydrogenase-3-type hydrogenases.

In addition to these four subunits, the *M. thermoautotrophicum eha* operon encodes three polyferredoxins and 11 conserved hypothetical subunits—ten predicted integral membrane proteins and one hydrophilic protein. All of these proteins are expressed and, therefore, thought to be functional subunits of the *M. thermoautotrophicum* Eha hydrogenase complex (Tersteegen and Hedderich 1999), although direct experimental data are lacking. The remaining proteins suggested by the transcriptional data have homologs in other, unrelated systems (not hydrogenases) and are located in the downstream region of the operon not tightly linked to the rest, thus precluding unambiguous assignment.

None of the 11 conserved hypothetical subunits are found in any experimentally characterized membrane-bound [NiFe]-hydrogenases (other hydrogenase-3-type hydrogenases such as Ech and Ecb have other, unrelated additional subunits). They are conserved only in four complete genomes of closely related Euryarchaeota (*M. thermoautotrophicum, Methanocaldococcus jannaschii, Methanopyrus kandleri* and *Methanococcus maripaludis*) and in *Methanobacterium thermoformicicum* (not a complete genome). Genome context of the corresponding genes in these organisms is also conserved, with the exception of the EhaI (PIRSF036537), which occurs only in *M. thermoautotrophicum* and *M. thermoformicicum*, and EhaK (PIRSF036538), which is missing in *M. kandleri* (they are replaced in the operons by unrelated membrane proteins). Based on these data, the corresponding eleven protein families were annotated as subunits of the multisubunit membrane-bound [NiFe]-hydrogenase Eha (Table 1). (Alternatively, they may encode components of a membrane-bound complex that couples hydrogenase activity to a process that is specific for methanogens, such as methanogenesis itself and/or some kind of electron transfer.) Thus, genome context, sequence similarity and phylogenetic profile collectively allow us to predict the function of the *M. jannaschii, M. kandleri* and *M. maripaludis* protein members of these families.

### Web-Based Access to PIRSF Protein Families

PIRSF protein families reflect evolutionary relationships, and function often follows along the family and/or subfamily lines. For a biologist seeking to collect and analyze information about a protein, matching a protein sequence to a curated protein family provides a tool that is usually faster and more accurate than searching against a protein database. The PIRSF family classification system is freely accessible from the PIR web site at http://pir.georgetown.edu/pirsf/ for researchers to explore protein functional and evolutionary relationships.

The classification and expert annotation results are presented in PIRSF family reports (e.g., http://pir.georgetown.edu/cgi-bin/ipcSF?id=PIRSF000 186), with summaries organized in several sections: (i) *general information*: PIRSF number and general statistics (family size, taxonomy range, length range, keywords), as well as additional annotation for curated families, such as family name, bibliography, family description, representative and seed members, and domain architecture; (ii) *membership*: lists of all members separated by major kingdoms and members from model organisms; (iii) *function, structure, and family relationship*: enzyme classification (EC, http://www.chem.qmw.ac.uk/iubmb/enzyme/), structure hierarchy (SCOP, Andreeva et al 2004), gene ontology (GO, Harris et al 2004), as well as family relationships at the full-length protein, domain, and motif levels with direct mapping and links to other family, function, and structure classification schemes, such as Pfam and InterPro (Mulder et al 2003); and (iv) *graphical display*: domain architecture of seed members or all members. The curation status of a PIRSF family is indicated as “full” (currently 3,963 PIRSFs), “preliminary” (4,516 PIRSFs), or “uncurated” (25,271 PIRSFs) meaning fully curated with optional description and bibliography, partially curated with membership and domain architecture, or automatically classified and not yet curated, respectively. The curated families, each with a unique ID and family name, are labeled with an evidence tag of “validated” to indicate those containing at least one member with experimentally-validated function, “predicted” for families whose functions are inferred computationally based on sequence similarity and/or functional associative analysis, or “tentative” to indicate cases where experimental evidence is not decisive. PIRSF protein family reports provide supporting evidence for both experimentally validated and computationally predicted annotations.

The PIRSF reports connect to several graphical viewers, including: (i) PIR taxonomy tree browser from the “Taxonomy Range” field, which displays the taxonomy distribution of all family members and the phylogentic pattern of members in complete genomes; (ii) PIR interactive alignment and tree viewer from the “Alignment and Tree” field, which displays ClustalW multiple alignment and neighbor-joining tree, together with a protein annotation table, all dynamically generated from seed members of curated families; and (iii) PIR DAG browser from the “PIRSF Hierarchy” field, which displays the PIRSF family hierarchy with Pfam domain superfamilies and protein membership in a network structure.

More than 20 PIRSF fields are searchable, including database unique identifiers (eg PIRSF ID, Pfam ID and PDB ID) and annotations (eg PIRSF family name, description, GO term and length). For example, one can identify all PIRSFs sharing one or more Pfam domains based on Pfam ID or name, or identify all PIRSFs in a SCOP fold superfamily based on SCOP fold name. The search results are returned in a summary table, listing each PIRSF with its ID, name, domain architecture and other major attributes, which can be tailored using different display options. The PIRSFs in the result summary table can be selected for further browsing and analysis, including multiple sequence alignment, taxonomy distribution and domain display. For PIRSF classification of a query protein, the “PIRSF Scan” program allows one to identify best-matched PIRSF families based on HMMER match (Eddy 1995) against both full-length and domain HMM models of all fully-curated PIRSF families.

## Using PIRSF for Protein Functional and Evolutionary Analysis

Protein classification facilitates systematic sequence and functional analysis of groups of proteins, allowing one to draw conclusions about protein evolution. In particular, PIRSF families that are fully curated based on integrative sequence and functional analysis and literature review allow the user to proceed directly to advanced comparative studies, for example, to detect *functional convergence* and *functional divergence.* There are different approaches for comprehensive studies of protein evolutionary and functional relationships. Functional convergence was investigated by Galperin et al (1998) by systematically identifying groups of enzymes with the same activity (as indicated by enzyme classification (EC) numbers in NCBI protein sequence databases) but unrelated sequence and structures. The PIRSF approach is to use annotated, evolution-based protein classification to assess for biological functions of the corresponding proteins. This would allow us to address a broad spectrum of protein groups, including proteins with non-enzymatic functions and enzymes not yet covered by the EC system. The sections below describe, with illustrative examples, how curated PIRSF families can be used to study the relationship between sequence evolution and protein function.

### Functional conservation in related proteins across taxonomic groups

**Figure 2:**
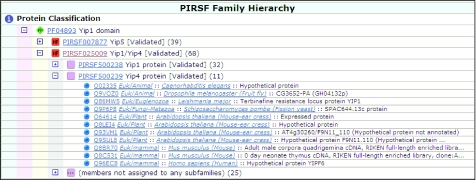
The PIRDAGbrowser view displaying PIRSF025009 family hierarchy with Yip1 and Yip4 subfamilies and protein membership.

Many PIRSF families cover broad taxonomic ranges and can be used to infer function of unknown proteins based on characterized homologs across different taxonomic groups as shown in the example of the PIRSF025009 family (Yipl/Yip4 [Validated]) and its two subfamilies—PIRSF50023 8 (Yipl [Validated]) and PIRSF500239 (Yip4 [Validated]). As shown in the DAG browser view of the PIRSF025009 family hierarchy (Figure 2), both subfamilies contain proteins from broad taxonomic divisions of fungi, animals and plants. Experimental evidence indicates that the yeast and mammalian members interact with Rab GTPases. These protein members are localized to the Golgi membrane and possibly to the endoplasmic reticulum (Heidtman et al 2003); therefore, they may function in the recruitment of Rab proteins from the cytosol to the membranes (Shakoori et al 2003). Their homologs in nematodes, insects and plants have no available experimental data. However, on the basis of sequence conservation, including the predicted 5 transmem-brane domains and the Pfam Yipl domain, it is likely that these proteins can also function as Rab-interacting proteins and may be involved in a similar biological process.

### Functional conservation and specialization in multi-domain proteins

An individual protein domain may fulfill the same biochemical role in various physiological systems or in different taxonomic groups; on the other hand, the combination of multiple domains may confer functional specialization unique to each particular system. The PIRSF classification system provides a convenient overview and comparison of protein families that share one or more Pfam domains. The domain architecture of curated families is represented in order from N- to C- terminus and may include functional Pfam domains that are not detected in all members using the default Pfam threshold values.

**Figure 3:**
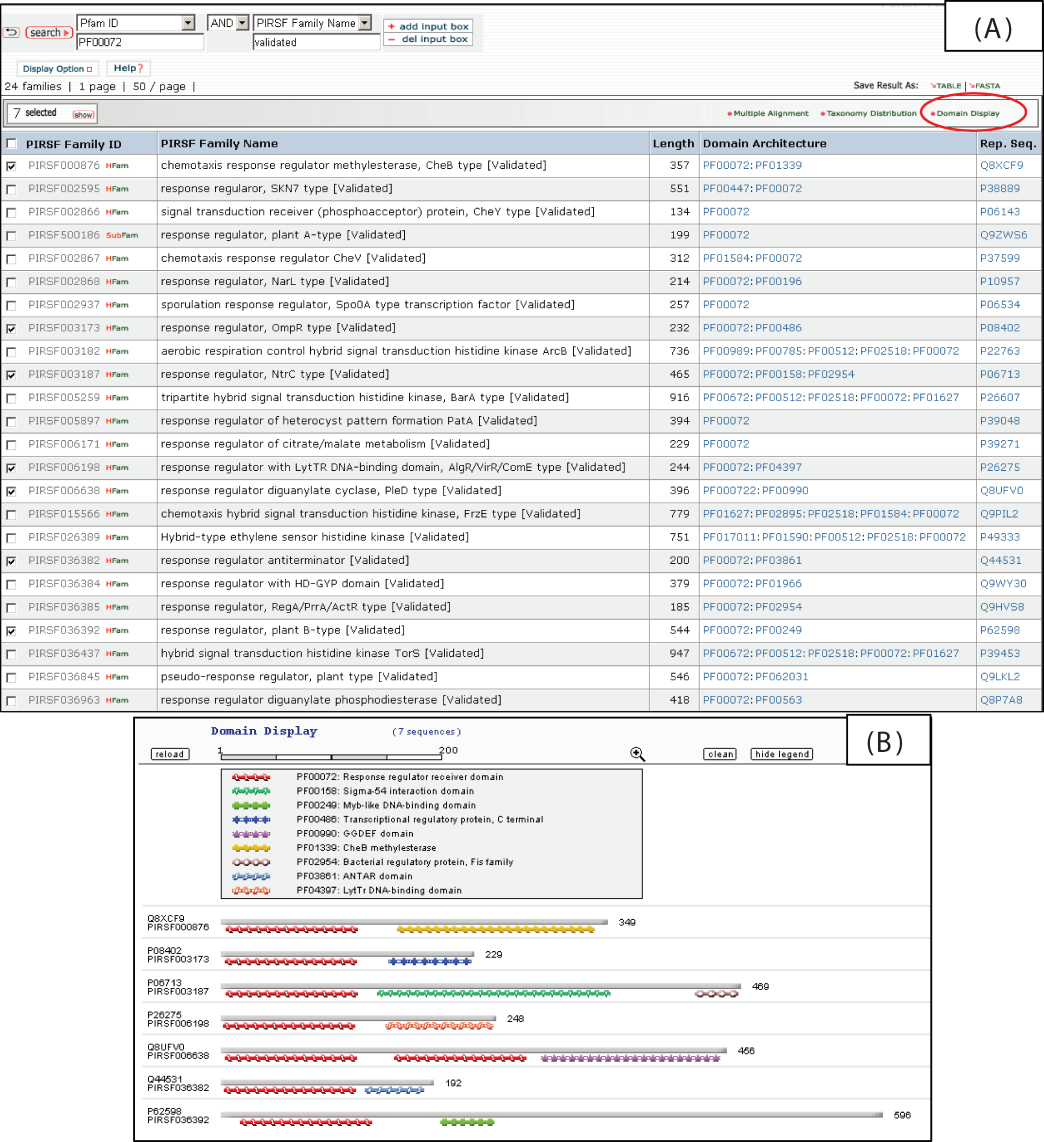
(A) Selected PIRSF response regulator families with CheY-like phosphoacceptor domain (PF00072); (B) domain display of the selected PIRSF families

The relationship between domain architecture and function can be illustrated by the various types of response regulators that share the CheY-like phosphoacceptor domain (PF00072) and are involved in signal transduction by two-component signaling systems. These response regulators usually consist of an N-terminal CheY-like receiver domain and a C-terminal output (usually DNA-binding) domain (Stock et al 2000). In addition to the “classical” well-known response regulators (eg PIRSF003173 with the winged helix-turn-helix DNA-binding domain), bacterial genomes encode a variety of response regulators with other types of DNA-binding domains (eg PIRSF006198, PIRSF036392), RNA-binding domain (PIRSF036382), or enzymatic domains (eg PIRSF000876, PIRSF006638), or a combination of these types of domains (eg PIRSF003187). Figure 3 shows a partial summary table that lists several PIRSF families that contain PF00072 from a text search using “Pfam ID” *PF000702* and “PIRSF name” *Validated* (Figure 3A) and the domain architecture of selected PIRSF families displayed using the “Domain Display” option (Figure 3B).

As revealed by the domain architecture and functional annotations of these PIRSF families, the presence of a unique output domain in response regulator families often signifies their involvement in a distinct regulatory pathway. For example, members of PIRSF006198 contain the LytTR DNA-binding domain and control the genes involved in biosynthesis of extracellular polysaccharides and bacteriocins, and in expression of exoproteins, including toxins, fimbriation, and quorum sensing (Nikolskaya and Galperin 2002). B-type plant response regulators (PIRSF036392) contain a eukaryotic Myb-like domain not found in prokaryotes, which is considered a multifunctional domain responsible for both nuclear localization and DNA binding (Hosoda et al 2002). Thus, B-type plant response regulators are a plant-specific evolutionary “innovation”, combining the receiver domain with a eukaryote-specific output domain and are involved in plant-specific signaling pathways, such as those mediated by ethylene or cytokinins. The RNA-binding transcription anti-termi-nator domain in PIRSF036382 confers the ability to prevent premature termination by interacting with the nascent mRNA upstream of the terminator (Wilson et al 1996; Shu and Zhulin 2002). Chemo-taxis response regulator methylesterases (CheB, PIRSF000876) contain a CheB methylesterase domain and are involved in chemoreceptor modification in bacterial chemotaxis (Djordjevic and Stock 1998). Members of PIRSF006638 contain GGDEF (signal transduction diguanylate cyclase) as an enzymatic output domain and act as regulators in pathways mediated by cyclic diguanylate, a novel global second messenger in bacteria (Ausmees et al 2001; Romling et al 2005).

### Functional divergence in closely related protein groups

In the PIRSF classification system, subfamilies are created to reflect functional specialization in homeo-morphic families. In many cases, functional divergence observed in the subfamilies are in fact completely new biological functions, as illustrated in the example of PIRSF000886 (metallophosphoesterase-fold protein, Vps29 type [Validated]). The family includes both prokaryotic and eukaryotic proteins that share the metal binding motif found in many other phosphoesterases. The PIRSF000886 family members, however, do not have the conserved catalytic His residue in the GNH[DE] portion of the common consensus pattern DXH(X25)GDXXD(X25)GNH[DE] (Zhuo et al 1994). As shown in the interactive tree and alignment view of the PIRSF000886 family (Figure 5), the His (H) residue is replaced with a Cys (C) or Asn (N) in most prokaryotic members, although these proteins still coordinate two divalent cations and have phosphodiesterase activity in vitro (Chen et al 2004; Kuznetsova et al 2005). The eukaryotic members, on the other hand, have no detectable phosphoesterase activity in vitro, while coordinating metals in a similar manner. Instead, this fold acts as a protein interaction scaffold for retromer assembly (Collins et al 2005). Therefore, these eukaryotic proteins were grouped and curated as a subfamily, PIRSF500276 (vacuolar protein sorting 29 [Validated]).

**Figure 4:**
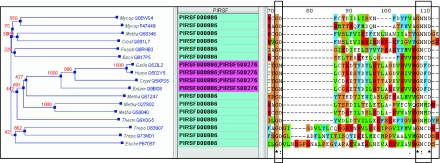
The PIR tree and alignment view of PIRSF000886 metallophosphoesterase-fold proteins, showing sequence variation of the Vps29 subfamily

Members of this subfamily are involved in vesicle-mediated transport (Seaman et al 1998; Arighi et al 2004; Seaman 2004) and have been shown to be part of the retromer complex, a pentameric membrane-associated protein complex that mediates in-tracellular recycling of receptors that sort vacuolar/lysosomal hydrolases. The retromer consists of two sub-complexes (Seaman et al 1998; Haft et al 2000): (1) Vps35/Vps29/Vps26 selects cargo for retrieval via binding of Vps35 to the cytosolic domain of the receptor, and (2) Vps5p(Snxl)/Vpsl7(Snx2) assembles onto the membrane to promote vesicle formation. The crystal structure of the human retromer subunit Vps29 shows that it has structural similarity to its prokaryotic counterparts, but a significant difference in the metal binding motif as well as the catalytic residue may explain the lack of activity. The human Vps29 bridges the metal ion through Asn (N) 39 instead of the Asp in prokaryotic phosphodiesterases, and uses Asp (D) 62 for metal binding instead of the Asn (N) in others. Finally, a conserved Phe (F) is located at the expected catalytic site (Figure 4). The subfamily reflects a subgroup of the homeomorphic family that has evolved very different biological functions in a different biological process from the typical metallophosphoesterases.

### Functional convergence of evolutionary unrelated proteins

**Figure 5:**
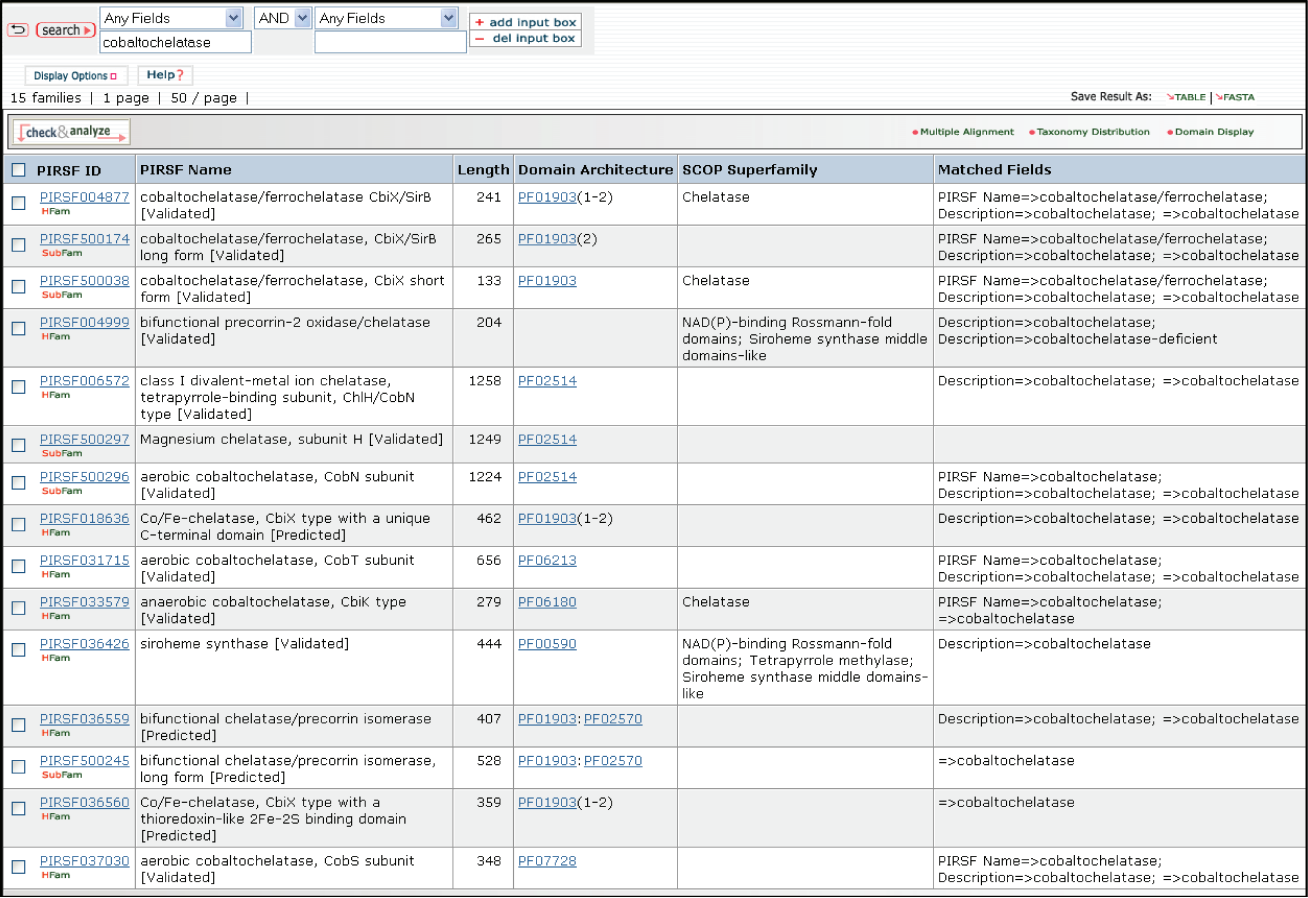
Functional convergence of cobaltochelatases in evolutionary unrelated PIRSF families that do not share common domain architecture or SCOP fold. Note that the number of PIRSF entries retrieved in this or any other search may change due to the addition of new annotations.

Combined information on curated PIRSF families, domain architecture and SCOP fold superfamily often reveals interesting information, such as functional convergence of evolutionarily unrelated proteins, as illustrated in the *cobaltochelatase* example. Figure 5 shows a list of homeomorphic families (HFam) and subfamilies (SubFam) retrieved by a text search using “Any Field” *cobaltochelatase.* The PIRSF view offers comprehensive information on the evolutionary and functional groups of cobaltochelatases and their properties, rather than just a list of all entries annotated as “cobaltochelatases” as do traditional protein databases. PIRSF004877, PIRSF018636, PIRSF036559, PIRSF036560 share the same Pfam domain—PF01903, the CbiX domain characteristic of class II chelatases—as reflected by the domain architecture column and the DAG browser view (HFam button). While members of these four families are under the same Pfam domain family, PIRSF classification view reflects evolutionary subgroups within this Pfam group and allows functional distinction among them. For example, it separates proteins with cobaltochelatase/ferrochelatase activity (PIRSF004877) from those with bifunctional chelatase/precorrin isomerase activity (PIRSF036559).

In addition, the ability to display customizable columns adds to the integrative view of the families. This is illustrated by the display of domain architecture column along with the SCOP Superfamily column, which reveals that PF01903 and another Pfam domain, PF06180 (CbiK, PIRSF033579) share the same SCOP fold superfamily (chelatase), indicating that these proteins may have arisen from a common ancestor (Schubert et al 1999).

The analysis and examination of highly curated PIRSF reports (especially descriptions and bibliography) further provides a more complete picture of the cobaltochelatase enzymes and the relationship of the class II chelatases to other chelatases, as summarized below. Tetrapyrrole biosynthetic chelatases fall into three classes, which are evolutionary unrelated based on sequence and structures (Brindley et al 2003). Members of all three classes include cobaltochelatases and ferrochelatases involved in biosynthesis of siroheme and cobalamin, which are related macrocyclic structures derived from uroporphyrinogen III.

Class I chelatases require three subunits for activity and utilize ATP. Aerobic cobalt chelatase consists of three subunits, CobT (PIRSF031715), CobN (PIRSF006572, subfamily PIRSF500296) and CobS (PIRSF037030) (Debussche et al 1992; Heldt et al 2005). Class II chelatases are defined as homomeric monofunctional chelatases that do not require ATP for catalysis (Brindley et al 2003). Class II chelatases include cobaltochelatases CbiK (Raux et al 1997) (PIRSF033579), SirB (Raux et al 2003) and CbiX (Brindley et al 2003) (PIRSF004877). It has been suggested that class II chelatases may have evolved from a primordial CbiX type of enzyme, which is thought to correspond to the short form of CbiX (subfamily PIRSF500038), without domain duplication (Brindley et al 2003).

A third, recently defined class of chelatases includes CysG (PIRSF036426) and Met8p (PIRSF004999), which are multifunctional proteins, acting as dehydrogenases and chelatases (Brindley et al 2003). They are able to chelate both Fe^2+^and Co^2+^ and can, therefore, function in both siroheme and cobalamin biosynthesis. In many organisms, precorrin-2 oxidase/ferrochelatase is fused with the uroporphyrin-III C-methyltransferase to form a two-domain siroheme synthase (PIRSF036426). PIRSF004999 represents a stand-alone form of precorrin-2 oxidase/ferrochelatase (eg yeast Met8p), which corresponds to the N-terminal domain of siroheme synthase. As with the class II chelatases, these proteins are homodimers and do not require ATP for activity. However, they share no structural similarity with the class II chelatases, and likely have arisen by the acquisition of a chelatase function within a dehydrogenase catalytic framework (Schubert et al 2002). The cobaltochelatase example thus illustrates functional convergence revealed by similar activities of characterized members of evolutionarily unrelated PIRSF families.

### Acquisition of function via horizontal gene transfer

**Figure 6:**
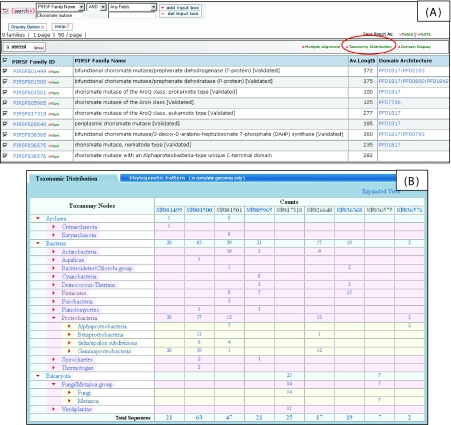
(A) Chorismate mutase-related PIRSF families and (B) taxonomic distribution of family members

Taxonomic analysis of related PIRSF families can reveal potential horizontal gene transfer that may have functional implications, as shown by the example of nematode chorismate mutase (PIRSF036575). Chorismate mutase (CM) (EC 5.4.99.5) catalyzes the reaction at the branch point of the biosynthetic pathway leading to the three aromatic amino acids, phenylalanine, tryptophan and tyrosine. It is part of the shikimate pathway, which is present only in bacteria, archaea, fungi, and plants. The taxonomic browser view of the nine chorismate mutase-related PIRSF families (Figure 6), however, reveals that PIRSF036575 is an animal-specific family, or more specifically (as revealed by expanding the Metazoa node down to the leaf nodes), is found in a specialized group of plantpathogenic nematodes. Nematode CMs are produced in the esophageal glands and secreted into the plant and are thought to be involved in virulence. They appear to function within the plant cell to manipulate the plant’s shikimate pathway, which controls plant cell growth, development, structure, and pathogen defense (Bekal et al 2003).

Sequence similarity analysis shows that the nematode CM is most closely related to periplasmic CM (PIRSF026640). Periplasmic CM is a subclass of the AroQ class CM, and is twice the size of cytoplasmic AroQ protein due to a unique C-terminal domain of unknown function (Calhoun et al 2001). Members of the periplasmic CM family may be involved in pathogenicity, as most members are pathogenic bacteria. This opens a possibility that chorismate mutases may have been acquired in plant-pathogenic nematodes as a result of horizontal gene transfer from bacteria with the conservation of pathogenicity-related function.

## Conclusions

PIRSF classification, which considers both full-length similarity and domain architecture, discriminates between single- and multi-domain proteins, showing functional differences associated with the presence or absence of one or more domains. Furthermore, specific biological functions (as opposed to generic biochemical functions) can seldom be inferred solely from the generic functions of the constituent domains, and proteins with different biological functions may have similar domain organization. Therefore, full-length protein functional annotation, based on homeomorphic protein families (sharing the same domain architecture and often the same biological function of the whole protein) and subfamilies (sharing the same function), is also important for providing the high-quality functional annotation.

The PIRSF classification provides family-based annotation for individual protein members. This annotation method has advantages over traditional “genome-by-genome” or “protein-by-protein” annotation, especially when coupled with the PIR name rules and site rules for accurate and consistent transfer of annotations from the corresponding PIRSF families and subfamilies (Natale et al 2005).

Coupling protein family classification and data integration allows associative studies of protein sequence, function, and structure. Domain-based or structural classification-based searches allow identification of protein families sharing domains or structural fold classes. Functional convergence and functional divergence are revealed by the relationships between protein family classification and curated family names and functions. With the underlying taxonomic information, protein families that occur in given lineages can be identified. The systematic approach for protein family curation using integrative data leads to novel predictions and functional inferences for uncharacterized “hypothetical” proteins, and to detection and correction of genome annotation errors. Such studies may serve as a basis for further analysis of protein functional evolution.

## Figures and Tables

**Table 1:** Genome context of [NiFe]-hydrogenase-3-type complex Eha in completely sequenced Euryarchaeotic genomes. The first 15 genes in the operon are shown

PIRSF ID and Name	Mt* Gene	Mk* Gene	Mj* Gene	Mm* Gene
PIRSF005019: [NiFe]-hydrogenase-3-type complex Eha, membrane protein EhaA	MTH384	MK0477	MJ0528	MMP1448
PIRSF019706: [NiFe]-hydrogenase-3-type complex Eha, membrane protein EhaB	MTH385	MK0476	MJ0527	MMP1449
PIRSF036534: [NiFe]-hydrogenase-3-type complex Eha, membrane protein EhaC	MTH386	MK0475	MJ0526.1	MMP1450
PIRSF006581: [NiFe]-hydrogenase-3-type complex Eha, membrane protein EhaD	MTH387	MK0474	MJ0526	MMP1451
PIRSF036535: [NiFe]-hydrogenase-3-type complex Eha, membrane protein EhaE	MTH388	MK0473	MJ0525	MMP1452
PIRSF019373: [NiFe]-hydrogenase-3-type complex Eha, membrane protein EhaF	MTH389	MK0472	MJ0524	MMP1453
PIRSF019136: [NiFe]-hydrogenase-3-type complex Eha, membrane protein EhaG	MTH390	MK0471	MJ0523	MMP1454
PIRSF036536: [NiFe]-hydrogenase-3-type complex Eha, membrane protein EhaH	MTH391	MK0470	MJ0522	MMP1455
PIRSF036537: [NiFe]-hydrogenase-3-type complex Eha, membrane protein Ehal	MTH392	--	--	--
PIRSF000215, subfamily PIRSF500037: [NiFe]-hydrogenase-3-type complex, membrane subunit C/D/J	MTH393	MK0468	MJ0520	MMP1457
PIRSF036538: [NiFe]-hydrogenase-3-type complex Eha, membrane protein EhaK	MTH394	--	MJ0519	MMP1458
PIRSF004953: [NiFe]-hydrogenase-3-type complex Eha, membrane protein EhaL	MTH395	MK0466	MJ0518	MMP1459
PIRSF005292: [NiFe]-hydrogenase-3-type complex Eha, hydrophilic subunit EhaM	MTH396	MK0465	MJ0517	MMP1460
PIRSF002913, subfamily PIRSF500034: [NiFe]-hydrogenase-3-type complex, small subunit	MTH397	MK0464	MJ0516	MMP1461
	MTH398	MK0463	MJ0515	MMP1462

* Mt, *Methanobacterium thermoautotrophicum* str. Delta H; Mj, *Methanocaldococcus jannaschii* DSM 2661; Mk, *Methanopyrus kandleri* AV19; Mm, *Methanococcus maripaludis* S2.

-- Genes encoding proteins not homologous to the *Methanobacterium thermoautotrophicum* counterparts, and therefore not members of the listed PIRFs.
